# Laser and light-based therapies combined with topical agents for melasma: A systematic review and meta-analysis

**DOI:** 10.1097/MD.0000000000046579

**Published:** 2026-01-09

**Authors:** Risha Fillah Fithria, Yehuda Tri Nugroho Supranoto, Zhaoqian Liu, Jinfu Peng

**Affiliations:** a Department of Clinical Pharmacy, Xiangya School of Pharmaceutical Sciences, Central South University, Changsha, China; b Faculty of Pharmacy, Universitas Wahid Hasyim, Semarang, Middle Java, Indonesia; c Bioinformatics Research Center, Institute of Bioinformatics (INBIO), Malang, Indonesia; d Department of Clinical Pharmacology, Hunan Key Laboratory of Pharmacogenetics, Xiangya Hospital, Central South University, Changsha, China.

**Keywords:** efficacy, laser light, melasma, safety, topical agents

## Abstract

**Background:**

This comprehensive review aimed to evaluate the efficacy and safety of combining laser or light therapy with topical agents for melasma treatment.

**Methods:**

A comprehensive search was conducted utilizing PubMed, NCBI, Google Scholar, ScienceDirect, Wiley, Embase, and the Cochrane Library. The search aimed to identify pertinent studies from the database’s inception until August 31, 2024. This research encompassed 11 randomized controlled trials including 461 people. The melasma area and severity index and its modified variant (mMASI) were employed for outcome assessment. The Cochrane risk-of-bias tool was used to assess study quality. The GRADE (grading of recommendations assessment, development and evaluation) approach was employed to assess the certainty of evidence. Sensitivity analyses were performed to evaluate the robustness of findings. Data were analyzed using RevMan 5.4.1.

**Results:**

Combination therapy demonstrated no significant benefit at 4 weeks, but statistically significant improvements at 8, 12, and 16 weeks (standardized mean difference [SMD]: −0.55; 95% confidence interval: −0.74 to −0.36; *P* < .00001), indicating a cumulative therapeutic effect. Moderate heterogeneity was observed (*I*^2^ = 64%). Sensitivity analysis excluding parallel-design studies (Souza, Bansal, Verma, Qu) confirmed the robustness of efficacy outcomes, with a similar effect size (SMD: −0.45) and reduced heterogeneity (*I*^2^ ≈ 41%). Safety analysis showed a significantly higher risk of adverse events in the combination group (OR: 8.96; 95% confidence interval: 3.71–21.64), mainly erythema and post-inflammatory hyperpigmentation. The certainty of evidence was rated as moderate for efficacy and very low for safety outcomes. Both were judged critically important (importance score 9 and 8, respectively) for clinical decision-making.

**Conclusion:**

These findings suggest that combination therapy can effectively enhance melasma treatment. However, careful patient selection and monitoring are crucial due to the risk of side effects. Further large-scale studies with standardized protocols are recommended to validate these findings and optimize treatment strategies.

## 1. Introduction

Melasma is a common facial skin condition that causes hyperpigmented macules. It is more common in women, especially those with darker skin, and is linked to hormonal fluctuations, sun exposure, and genetics.^[1,2]^ Melasma is caused by melanocyte melanin synthesis, inflammation, and vascular alterations, and can be aggravated by ultraviolet exposure.^[3,4]^ Melasma is characterized by epidermal, dermal, or mixed pigmentation levels, each with specific clinical characteristics and therapeutic needs. This condition is more common in locations with intense sunlight.^[4]^

Melasma can trigger significantly negative physiological and psychological effects. The leading cause of these effects is the chronic and often resistant nature of melasma, which makes it challenging to manage, and can heighten the associated physical and emotional burden.^[5,6]^

Various therapeutic modalities have been developed to treat melasma, including combination treatment that integrates laser- or light-based therapies with topical agents. Melasma laser and light treatments have many advantages over the traditional methods. Intense pulsed light (IPL) and fractional lasers diminish melasma pigmentation. Studies have shown that fractional lasers target melanin deposits and remodel collagen, thereby improving skin texture and tone.^[7]^ Combining laser- or light-based therapies with topical melasma medicines improves the therapeutic efficacy and patient outcomes. Laser therapies, such as fractional laser therapy, target melanin in the skin and reduce pigmentation. They may not address hormonal factors and inflammation, which cause melasma.^[8]^ Topicals, including hydroquinone, azelaic acid, and retinoids, decrease melanin formation and rejuvenate skin cells.^[9]^ Synergistic results can be obtained by combining these 2 modalities. Laser therapy reduces pigmentation, whereas topical medicines prevent recurrence and accelerate skin healing.^[10]^

Such combination techniques have been shown to improve treatment efficacy compared with monotherapy. Combining fractional laser treatment with topical medications improved melasma severity and patient satisfaction in 1 trial.^[11]^ Another study found that laser and topical therapies synergistically improved skin pigmentation.^[12]^ Despite these promising findings, there are still unresolved issues and controversies regarding their overall efficacy and safety. Some studies have shown minimal improvement or even worsening of melasma, particularly in individuals with darker skin types, due to post-inflammatory hyperpigmentation (PIH) risks.^[13]^ Additionally, inconsistent treatment protocols, variations in laser parameters, and differences in skin phototypes and topical agents used have led to considerable heterogeneity in outcomes.

These ongoing uncertainties highlight the need for a comprehensive synthesis of current evidence. Therefore, this systematic review and meta-analysis aims to clarify the clinical efficacy and safety of combining laser or light therapy with topical agents for melasma treatment based on data from randomized controlled trials (RCTs).

## 2. Materials and methods

The systematic review and meta-analysis were preregistered with PROSPERO under CRD42024556539 (refer to Appendix 1, Supplemental Digital Content, https://links.lww.com/MD/Q986 which displays the PROSPERO registration). The study followed the Preferred Reporting Items for Systematic Reviews and Meta-Analyses (PRISMA) 2020 recommendations.^[14]^

### 2.1. Search strategy

A systematic search was performed using PubMed, NCBI, Google Scholar, ScienceDirect, Wiley, Embase, and Cochrane Library. The search sought relevant studies from the database’s inception until August 31, 2024. Two independent reviewers, RFF and YTNS, conducted this search, and the supplementary data detail their methodologies (refer to Appendix 2, Supplemental Digital Content, https://links.lww.com/MD/Q985 which delineates the literature search approach). The reference lists of the included studies and pertinent systematic reviews were manually reviewed to identify additional eligible studies.

### 2.2. Study selection

RCTs examined the safety and efficacy of laser or light therapy with topical melasma medications to determine whether the selected studies met the criteria. Studies on pregnancy, breastfeeding, and attempts to conceive were excluded. Individuals with treatment-area skin concerns, recent chemical peels or laser treatments (within 3 months), or face depigmentation topical drugs (within 4 weeks) were excluded. The trial participants were healthy adults with symmetrical mild, moderate, or severe melasma on both sides of their faces and all Fitzpatrick skin phototypes. Dermatologists should confirm this diagnosis using specific instruments. Two independent reviewers, RFF and YTNS, screened the titles, abstracts, and entire text for eligibility. Discourse-resolved investigator disputes with consensus.

### 2.3. Outcome measures

The primary outcome was melasma severity improvement from baseline to follow-up, measured using the melasma area and severity index (MASI) score or its modified melasma area and severity index (mMASI). Secondary outcomes included adverse events (AEs).

### 2.4. Data extraction

The RFF and YTNS use a standardized data extraction form to gather baseline and outcome data independently. Consensus resolved these differences. Data from each study included the first author’s last name, year of publication, country of origin, study design, study duration, total number of participants, number of participants who completed the study, sex distribution, average age of participants, distribution by Fitzpatrick skin type, detailed descriptions of interventions (covering the experimental and control groups, treatment duration, and follow-up treatment), and outcome measures (MASI/mMASI). Data from several study groups were acquired using the Cochrane Handbook procedures.^[15]^ The data obtained for continuous outcomes comprised means, standard deviations, and sample sizes at both the baseline and follow-up stages. Dichotomous outcomes were analyzed by extracting the number of cases and total sample sizes.

### 2.5. Quality assessment

Two investigators (RFF and YTNS) independently evaluated the risk of bias using the revised Cochrane Risk of Bias Tool (RoB 2.0, version 2023, available at www.riskofbias.info).^[16]^ A discussion was conducted to resolve the discrepancies. The RCTs were assessed for bias and graded as low-, moderate-, or high-quality based on specific criteria. An RCT was classified as low quality if either randomization or allocation concealment had a high risk of bias.

### 2.6. Data synthesis and analysis

RCT data were processed using Microsoft Excel prior to the meta-analysis. The meta-analysis used RevMan (version 5.4.1) with random effects was used for the meta-analysis. SMD and risk ratio were used to depict pooled continuous and dichotomous data with 95% confidence intervals (CIs). We assessed the combined SMD using conventional guidelines to better understand the anticipated effectiveness: A number between 0.4 and 0.7 suggests a moderate influence, and >0.7 indicates a strong effect.^[15]^

This study used a meta-analysis to evaluate laser or light treatment in combination with topical medicines at various follow-up times and their adverse effects. Thus, the subgroup analyses depended on the intervention follow-up length and AE types. Heterogeneity was assessed using the *I*^2^ statistic. *I*^2^ statistics classified values from 0 to 40 as unimportant, 30 to 60 as moderate heterogeneity, 50 to 90 as substantial heterogeneity, and 75 to 100 as heterogeneity. The overall effect was tested for statistical significance using a *P*-value of <.05. The researchers examined funnel plots for small-study effects that could indicate publication bias.^[15]^

### 2.7. Certainty of evidence assessment

The certainty of the evidence was assessed using the GRADE approach, evaluating risk of bias, inconsistency, indirectness, imprecision, and publication bias. Outcomes were categorized as high, moderate, low, or very low certainty. Each outcome was also rated for its importance in clinical decision-making using a 9-point scale, with scores of 7 to 9 considered critically important. The GRADE assessment was conducted using the GRADEpro online platform (https://gradepro.org/).

### 2.8. Sensitivity analysis

Sensitivity analysis was conducted to assess the robustness of the primary outcome (efficacy). Studies using a parallel-group design (Souza, Bansal, Verma, and Qu) were excluded due to methodological variation. The results remained statistically significant, and heterogeneity decreased from 64% to ~41%, indicating that the findings were stable and not driven by study design differences. Sensitivity analysis was not performed for safety outcomes due to limited and inconsistent reporting of AEs across studies.

## 3. Results and discussion

### 3.1. Search results

**Figure 1.**
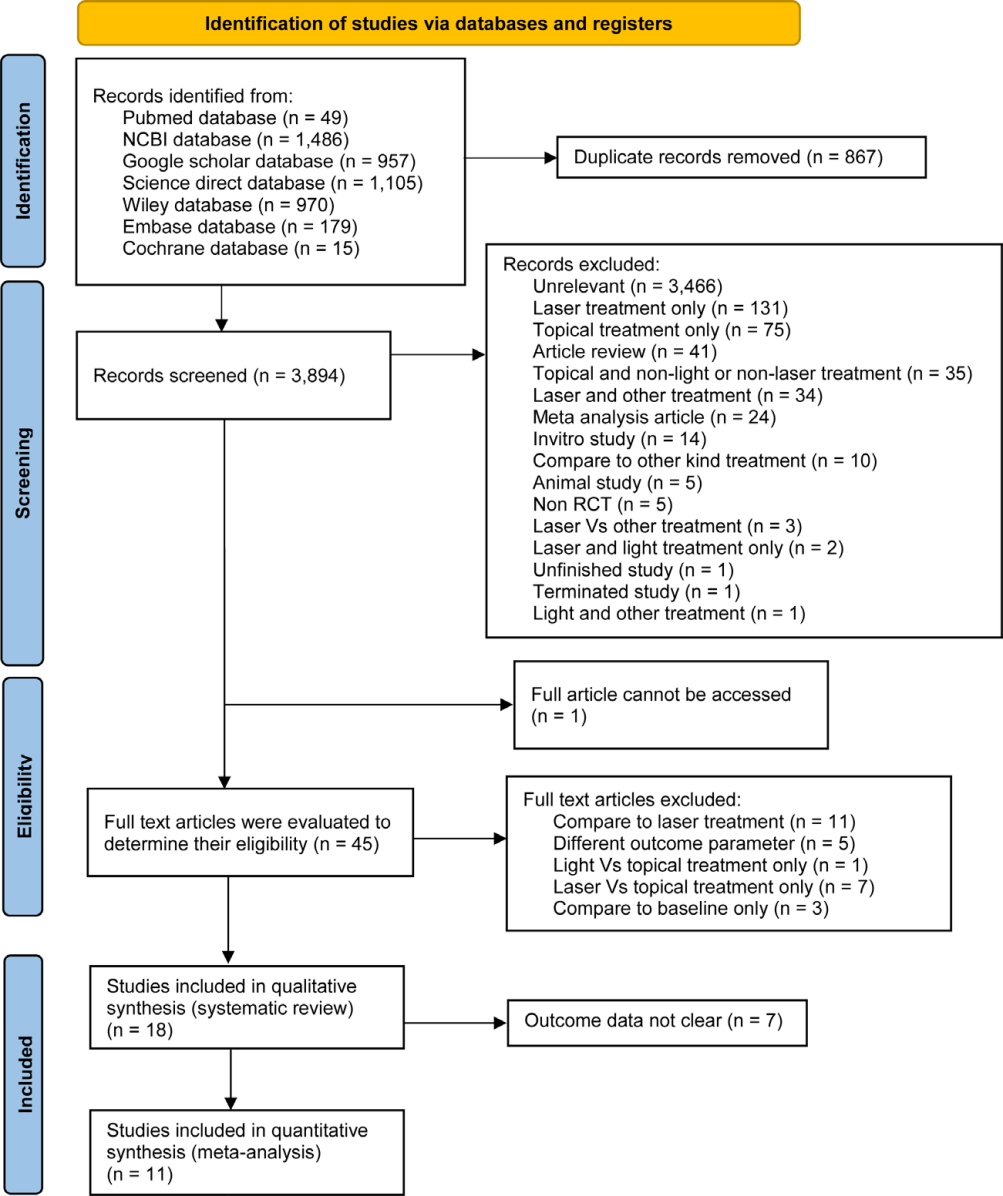
Preferred reporting items for systematic reviews and meta-analyses (PRISMA 2020). PRISMA = Preferred Reporting Items for Systematic Reviews and Meta-Analyses, RCT = randomized controlled trial.

A preliminary literature search yielded 3894 unique results. After screening the titles and abstracts, 3849 records were declared ineligible and were removed from the study. In addition, 1 article was unavailable. After thorough review of the text, 18 articles were included. Only 11 RCTs were suitable for meta-analysis, as 7 of the 18 had uncertain outcomes.^[17–27]^ Figure 1 illustrates the research selection process.

### 3.2. Study characteristics

These 11 trials included 461 patients with melasma from 8 nations. Brazilian, Indian, Chinese, South Korean, Egyptian, Iranian, Iraqi, and Thai studies have also been conducted. Four studies recruited men and women, and 7 exclusively recruited women. A total of 450 participants (97.61%) were female. The 24- to 44-year-olds had skin phototypes II–V. The attributes of this study are in Table 1.

**Table 1:** Characteristics of included studies.

Number	Included studies	Location	Study design	Sample (T/C)	Average age	Gender	Fitzpatrick skin type	Experiment group (EG)	Control group (CG)	Treatment	Follow-up	Outcome	Adverse events N (%)
1	Souza LF and Souza ST^[17]^	Brazil	Randomized controlled trial, parallel-group	62/50	44.3 ± 6.1 yr	58 F/4 M	Type II: 7 Type III: 21 Type IV: 26 Type V: 8	1. IPL in a single session 2. Hydroquinone 4%, tretinoin 0.05%, fluocinolone acetonide 0.01% once daily	Hydroquinone 4%, tretinoin 0.05%, fluocinolone acetonide 0.01% once daily	48 wk	4 wk 24 wk	MASI score	Mild erythema and pain Microcrust Burning sensation PIH (EG. = 3)
2	Bansal, C et al^[18]^	India	Randomized controlled trial, parallel-group	60/60	37.7 ± 6.63 yr	59 F/1 M	Type III: 1 Type IV: 31 Type V: 28	A = 1 B = 2 C = 1 and 2 1. Low-fluence Q-switched Nd:YAG laser (QSNYL) once weekly 2. Topical 20% of azelaic acid cream twice a day	Topical 20% azelaic acid cream twice a day	12 wk	6 wk 12 wk	MASI score	Erythema (EG = 1) Burning/stinging (EG = 1, CG = 1)
3	Verma, A and Agrawal, S^[20]^	India	Randomized controlled trial, parallel-group	60/51	31.05 ± 5.85 yr	59 F/1 M	Type II and III: 22 Type IV and V: 38	1. IPL 2 sessions at 2-wk intervals 2. Hydroquinone 2%, tretinoin 0.025%, fluocinolone acetonide 0.01% at night	Hydroquinone 2%, tretinoin 0.025%, fluocinolone acetonide 0.01% at night	12 wk	4 wk 8 wk 12 wk 16 wk	MASI score	Erythema (EG = 28, CG = 23) Telangiectasia (EG = 4, CG = 7) Burning (EG = 8) Urticaria (EG = 1)
4	Qu et al^[21]^	China	Randomized controlled trial, parallel-group	90/90	24–59 yr	All female	Type II: 14 Type III: 29 Type IV: 37 Type V: 10	1. Low-power fractional CO_2_ laser 3 wk intervals for 4 times 2. Tranexamic acid solution (30 mL, twice a day, for 10 min)	Tranexamic acid solution (30 mL, twice a day, for 10 min)	16 wk	4 wk 8 wk 12 wk 16 wk	MASI score	Red swelling, burning, different levels of pain (EG = 12, CG = 1) PIH (EG = 1)
5	Passeron, T et al^[22]^	South Korea	Randomized controlled trial, split-face study	18/17	41 yr (33–56 yr)	All female	Type II: 4 Type III: 8 Type IV: 6	1. Pulse-dye laser 3 sessions, 3 wk intervals 2. TCC (hydroquinone, 4%; tretinoin, 0.05%; and fluocinolone acetonide, 0.01%), once daily for 4 mo	TCC (hydroquinone, 4%; tretinoin, 0.05%; and fluocinolone acetonide, 0.01%) once daily for 4 mo	16 wk	16 wk	MASI score	A transient and mild irritation (EG side = 8, CG side = 8) PIH (EG side = 3)
6	Badawi, AM and Osman, MA^[23]^	Egypt	Randomized controlled trial, split-face study	32/30	37.87 ± 7.97 yr	All female	Type III: 11 Type IV: 12 Type V: 7	1. A fractional Er:YAG laser every 2 wk, 6 treatment sessions 2. HQ 4% cream twice daily	HQ 4% cream twice daily	10 wk	12 wk	MASI score	Erythema (EG side = 30, CG side = 5) Burning sensation (EG side = 7, CG side = 5) Superficial crusting (EG side = 30) Itching (EG side = 3, CG side = 3) Recurrence of melasma (both sides = 2)
7	Namazi, N et al^[24]^	Iran	Randomized controlled trial, split-face study	29/29	36.1 ± 8 yr	All female	Type II: 10 Type III: 9 Type IV: 10	1. The Er:YAG laser (erbium: yttrium–aluminum–garnet) monthly for 3 mo 2. HQ 4% cream once daily	HQ 4% cream once daily	12 wk	4 wk 8 wk 28 wk	MASI score	PIH (EG side = 5) Recurrence of melasma (EG side = 6)
8	Al-Dhalimi, MA and Yasser, RH^[25]^	Iraq	Randomized controlled trial, split-face study	31/25	36.08 ± 6.9 yr	All female	Type III: 5 Type IV: 20	1. Fractional Er:YAG laser, 6 laser sessions at 2 wk intervals 2. Kojic acid 1% and vitamin C 2% once daily	Kojic acid 1% and vitamin C 2% once daily	10 wk	12 wk 22 wk	MASI score	Mild erythema (EG side = 25, CG side = 25)
9	Nasimi, M et al^[26]^	Iran	Randomized controlled trial, split-face study	29/20	36.85 ± 6.18 yr	All female	Type II–IV with the number of patients of each type is not given	1. Er:YAG laser, 3 sessions every 4 wk 2. Kligman’s formula (4.0% hydroquinone, 0.1% dexamethasone, and 3% vitamin C) once daily	Kligman’s formula (4.0% hydroquinone, 0.1% dexamethasone, and 3% vitamin C) once daily	12 wk	24 wk	MASI score	Transient and mild erythema and scaling (EG side = 20) Worsening of melasma (EG side = 1, CG side = 1) Acne-like eruptions (EG side = 1, CG side = 1)
10	Beyzaee, AM. et al^[27]^	Iran	Randomized controlled trial, split-face study	30/27	38.37 ± 5.26 yr	All female	Not given	1. QSNd:YAG laser, 6 sessions with 2-wk intervals 2. Topical methimazole 5% once daily	Topical methimazole 5% once daily	12 wk	4 wk 8 wk 12 wk	mMASI score	Discomfort (EG side = 5, CG side = 6) Erythema (EG side = 11, CG side = 5)
11	Paichitrojjana, PS^[19]^	Thailand	Randomized controlled trial, split-face study	20/17	43.82 ± 6.31 yr	15 F/2 M	Type IV: 13 Type V: 4	1. The 1064 nm ps laser toning, 3 treatments for 4 wk intervals 2. Niacinamide 4% cream twice daily	Niacinamide 4% cream twice daily	12 wk	4 wk 8 wk 12 wk	mMASI score	Erythema, pruritus, burning sensation, desquamation, and popular eruption (EG side = 17)

CG = control group, HQ = hydroquinone, IPL = intense pulsed light, MASI = melasma area and severity index, mMASI = modified melasma area and severity index, PIH = post-inflammatory hyperpigmentation, QSNYL = Q-switched Nd:YAG laser, TCC = triple combination cream, YAG = yttrium aluminum garnet.

All the studies used topical medicines and lasers/lights. The investigations used an IPL, Q-switched Nd laser (QSNYL), fractional CO_2_ laser, pulse-dye laser, fractional erbium: YAG laser (FEYL), classic erbium: yttrium aluminum garnet (YAG) laser, and a picosecond laser.

Fixed-dose triple combination treatment with hydroquinone 4%, tretinoin 0.05%, fluocinolone acetonide 0.01%, azelaic acid 20%, hydroquinone (HQ) 4%, kojic acid 1% with vitamin C 2%, Kligmans’ formula with 4.0% hydroquinone, 0.1% dexamethasone, and 3% vitamin C; methimazole 5%; tranexamic acid (concentration not specified); and niacin-only tranexamic acid and methimazole were solutions, not creams. The intervention lasted for 10 to 48 weeks, with a maximum follow-up of 48 weeks. All the studies reported AEs. Four studies employed parallel designs,^[17,18,20,21]^ while the rest used a split-face. Three of the 11 RCTs declared that they were obtaining nonprofit sponsorship,^[19,24,26]^ while the others did not.

### 3.3. Quality assessment

**Figure 2.**
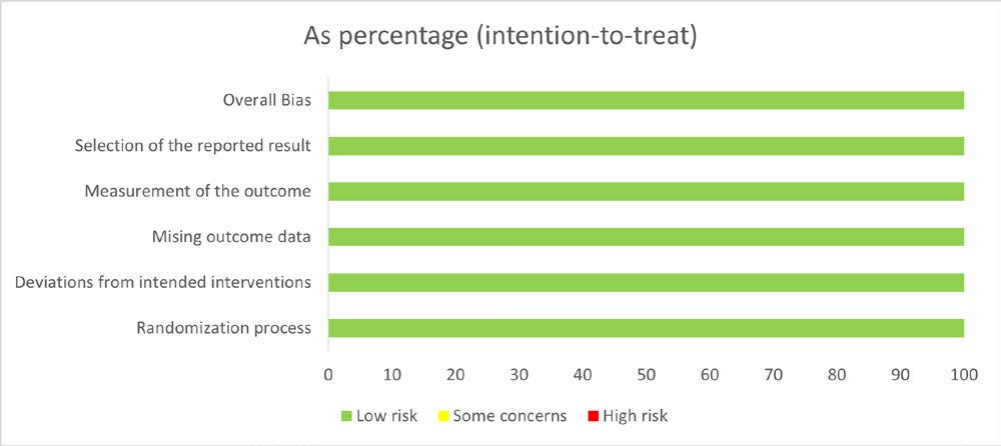
Evaluation of potential bias and study quality assessment of RCTs. RCT = randomized controlled trial.

Figure 2 details bias risk evaluation. The criteria deemed all studies high-quality. No research in the analysis revealed allocation concealment. Bias is unlikely in topical medications in laser or light treatment trials since allocation concealment is difficult. According to Naci et al, open-label studies with altered interventions are not inherently biased. Significant treatment variations may compromise blindness.^[28]^

### 3.4. Efficacy outcomes

The MASI and mMASI were the output parameters in the 11 randomized controlled studies. MASI comprehensively grades melasma and pigmentation. Consideration of facial participation and pigmentation intensity in various facial areas can thoroughly analyze the condition. Instead, mMASI improved the accuracy and precision of the original MASI. The mMASI preserves the MASI core, but alters the scoring criteria for clinical usage and covers skin type and subjective pigmentation assessment. Although scored differently, the MASI and mMASI strive to accurately assess melasma severity and treatment efficiency.^[29,30]^

**Figure 3.**
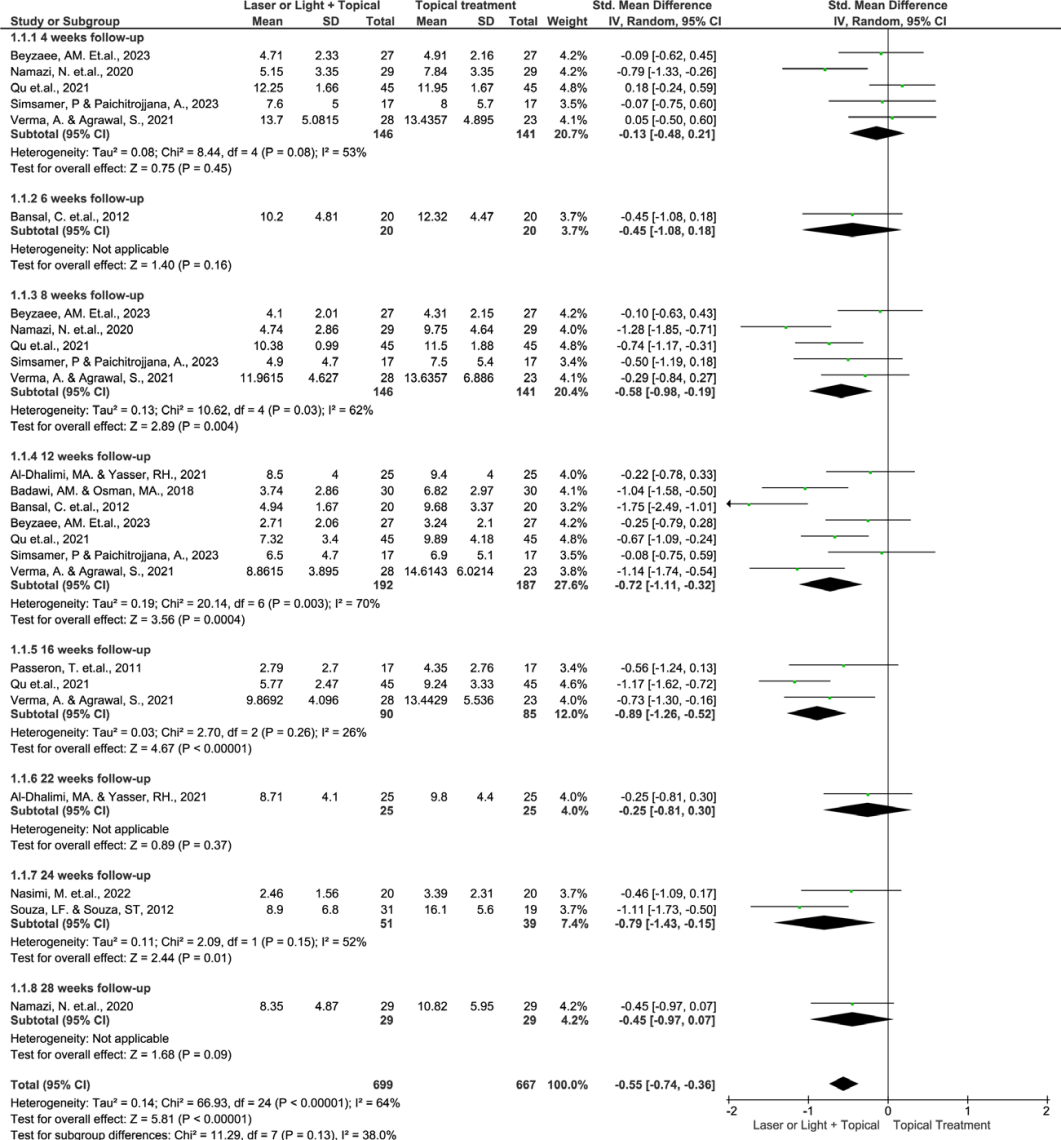
Forest plot of SMD for MASI/mMASI reduction in patients with melasma treated with laser/light and topical agents in RCTs stratified by follow-up time. MASI = melasma area and severity index, mMASI = modified melasma area and severity index, RCT = randomized controlled trial, SMD = standardized mean difference.

The 11 RCTs with MASI or mMASI outcomes included 461 participants. Pooled data showed various effects over time (Fig. 3). Four weeks later, the combination therapy group showed similar results. The therapies were similarly helpful at this early stage, as the SMD was −0.13 (95% CI: −0.48, 0.21; *P* = .45). Combination treatment had a minor but statistically significant advantage at 8 weeks (SMD: −0.58; 95% CI: −0.98, −0.19; *P* = .004). Laser or light treatment can improve the efficacy of topical remedies. SMDs of −0.72 and −0.89, after 12 and 16 weeks, respectively. These results demonstrate the cumulative effects of combination therapy.

The 22-week follow-up indicated a slight but insignificant improvement (SMD: −0.25; 95% CI: −0.81, 0.30; *P* = .37), suggesting that the treatment may be less effective. However, SMD of −0.79 (95% CI, −1.43, −0.15; *P* = .01) revealed combination treatment resurrection at 24-week follow-up. Therefore, this combined treatment was repeated. The 28-week follow-up result was not statistically significant (SMD: −0.45; 95% CI: −0.97, 0.07; *P* = .09), showing that treatment results fluctuated over time.

These findings demonstrate the benefits of laser or light therapy and topical melasma treatment. The decrease in melasma severity was greater in the combined therapy group than that in the topical therapy group. The total SMD was −0.55 (95% CI: −0.74, −0.36; *P* < .00001) indicated tremendous success with this technique. Combining these modalities may improve results, especially for melasma, which is notoriously difficult to treat and often resistant.^[31]^ The 4-week evaluation indicated no effect; therefore, the combined treatment took time to complete. The efficacy changes identified during subsequent evaluations underscore the necessity of ongoing assessment and therapy modifications to sustain and improve outcomes. Choosing a laser or light treatment for topical treatments requires consideration of the patient’s features and the skin phototype. This adjustable technique tailors combination therapy to each person’s demands, boosting the results.

Melasma characteristics are targeted by laser and light therapies. IPL and low-fluence Q-switched Nd:YAG lasers (QSNYL) target melanin, whereas fractional CO_2_ and Er:YAG lasers remodel collagen and remove pigmentation. The pulsed dye laser targets hemoglobin, which lowers melasma redness. Innovative 1064 nm ps laser toning removes melanin without heat. QSNYL requires weekly sessions, whereas fractional CO_2_, pulse-dye laser, and fractional Er:YAG require 2 to 3 weeks. Er:Monthly YAG laser sessions methodically reduce pigmentation, and laser or light therapy should depend on the skin type, melasma severity, and therapist’s experience.^[32,33]^

Melasma treatment involves the use of topical medications, lasers, and light. Fixed-dose triple hydroquinone (4%), tretinoin (0.05%), and fluocinolone acetonide (0.01%). Thus, it treats moderate-to-severe melasma. Skin creams containing 20% azelaic acid suppressed tyrosinase activity and minimized irritation. It also treats the sensitive skin and PIH. Antioxidants such as kojic acid and vitamin C block tyrosinase and replace hydroquinone. The Kligman formula is comprised of hydroquinone (4.0%), dexamethasone (0.1%), and vitamin C (3%) to increase its efficacy. Topical therapy, such as a fixed-dose triple combination cream with 4% HQ, can be administered daily or twice daily. The patient’s skin type, chemical tolerance, and melasma severity should guide the topical drug selection.^[34,35]^

Consider the patient’s characteristics and skin phototype when choosing a combination of laser or light therapy, and topical agents. Fitzpatrick IV–VI dark skin increases the risk of PIH. This risk can be reduced using lower-fluence laser settings, topical azelaic, or kojic acid. Sensitive skin may tolerate topical retinoids better than Kligman formula.^[34,35]^

These data imply that combined therapy is beneficial for treating melasma, especially in patients in whom topical therapy fails. This approach permanently resolves the pigmentation concerns.

A meta-analysis of 11 trials revealed a wide range of treatment effects with an *I*^2^ value of 64%. This value shows that research differences, and not sampling errors, explain 64% of the observed range in the findings. The substantial heterogeneity of the τ^2^ value of 0.14 and the *χ*^2^ statistic of 66.93 (df = 24, *P* < .00001) suggests inconsistent results from various studies. The *χ*^2^ value for subgroup differences was 11.29 (df = 7, *P* = .13), and the *I*^2^ score was 38.0%. The subgroup differences were not appear to be substantial. The research design, patient groups, and treatment techniques may explain the moderate variation observed in these studies.

**Figure 4.**
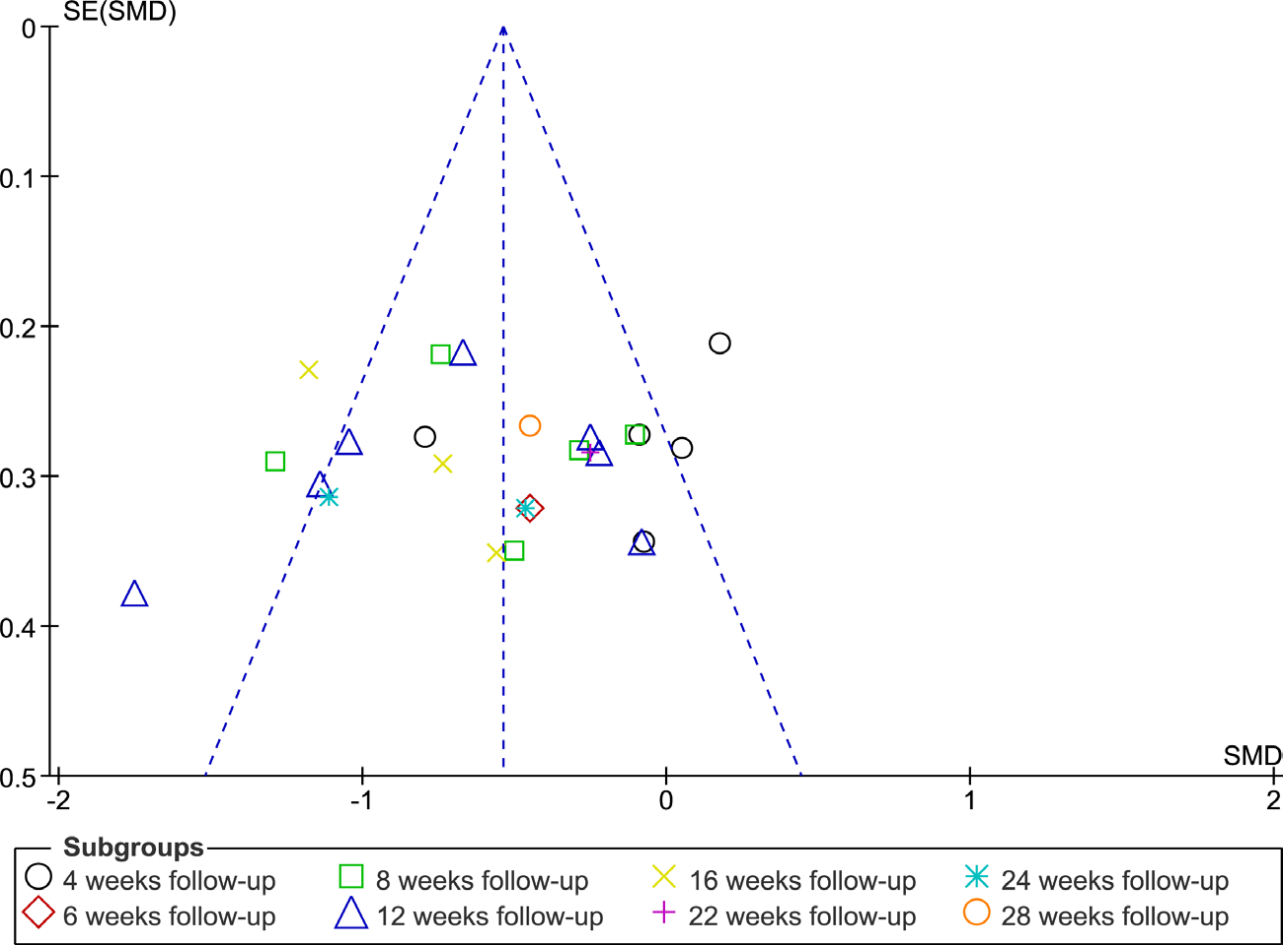
Funnel plots for MASI or mMASI outcome measures in RCTs. MASI = melasma area and severity index, mMASI = modified melasma area and severity index, RCT = randomized controlled trial, SE = side effects, SMD = standardized mean difference.

The funnel plot from 11 RCTs (Fig. 4) comparing laser or light treatment with topical medications in patients with melasma indicated asymmetry, notably in the smaller trials. This discrepancy may indicate heterogeneity or publication bias, because smaller positive studies are published more often. Heterogeneity may result from the trial design, demography, and treatment. Some trials with higher standard errors were outliers, suggesting a greater efficacy. Combination therapy was effective in more extensive studies; however, smaller trials with more significant results may distort the findings. Thus, combining laser or light and topical treatments should be interpreted cautiously, and well-designed large-scale studies are needed for a more conclusive conclusion.

### 3.5. Safety outcome

**Figure 5.**
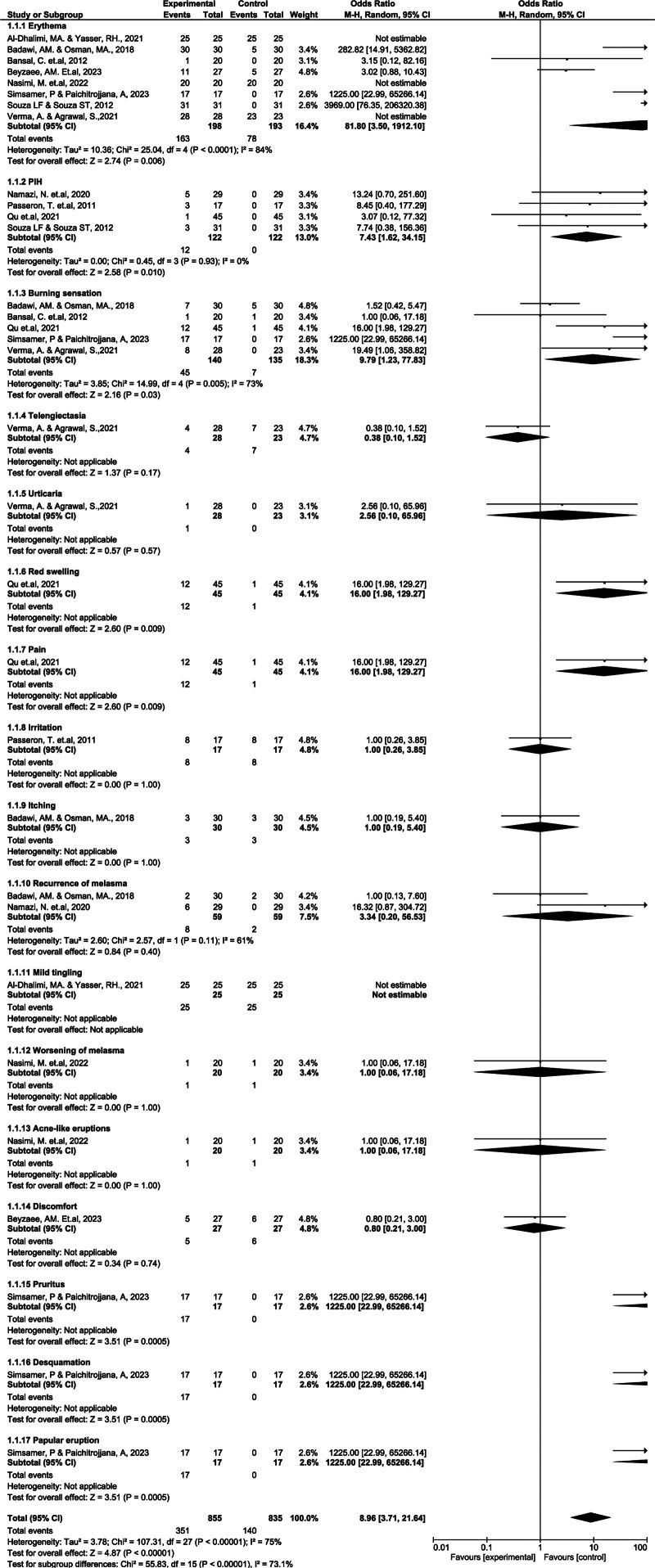
Forest plot of the risk ratio (RR) for safety outcomes in RCTs by adverse event type. RCT = randomized controlled trial, RR = risk ratio.

The safety analysis revealed a significantly higher risk of AEs in the combination therapy group (Fig. 5), with a pooled OR of 8.96 (95% CI: 3.71–21.64). The most frequently reported AEs were erythema and PIH, especially in patients with Fitzpatrick skin types III–V. The proposed mechanisms underlying these reactions include laser-induced epidermal injury, increased melanocyte activity, and inflammatory responses. Improper laser settings, lack of skin testing, or insufficient posttreatment care can exacerbate these effects. Although erythema is often transient and may indicate therapeutic response, PIH though expected can negatively affect patient satisfaction, particularly in darker skin types.^[36,37]^

Other common but generally self-limited adverse effects included burning sensations and discomfort, typically lasting a few days. These may be alleviated through adjunctive use of topical agents such as corticosteroids, niacinamide, or tranexamic acid, which possess anti-inflammatory and melanin-inhibitory properties. Patient education on strict photoprotection and gradual initiation of topicals can further reduce AE risk and severity.

Severe but less frequent events ike pruritus, desquamation, and papular eruptions were also noted (e.g., OR for pruritus: 1225.00), which may impact adherence and quality of life. Therefore, careful patient selection, tailored energy settings, and proactive AE management strategies are crucial. Future studies should focus on improving AE reporting consistency, identifying high-risk subgroups, and developing standardized safety protocols.

**Figure 6.**
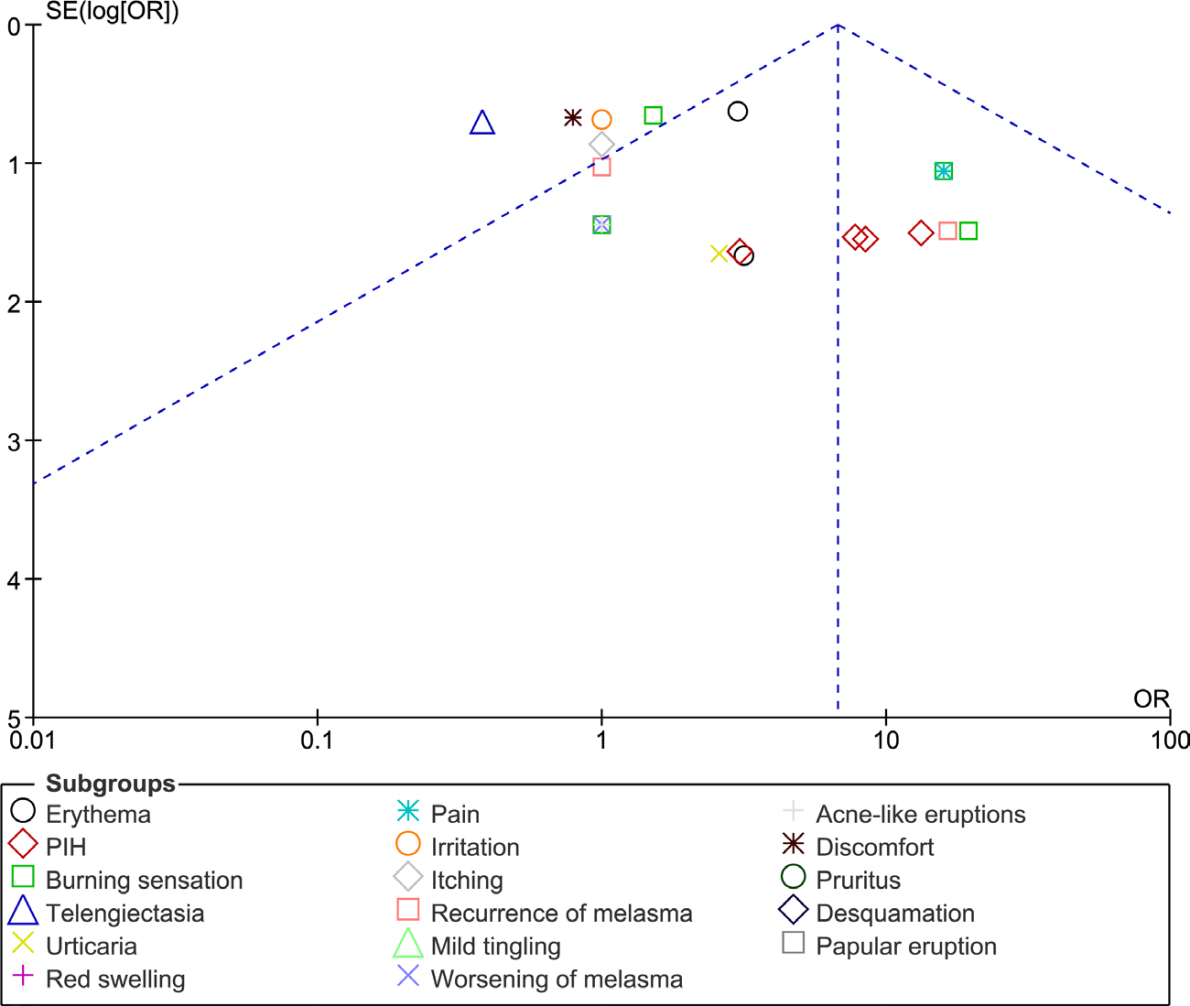
Funnel plots for adverse events (AE) in RCTs. AE = adverse event, OR = odds ratio, PIH = post-inflammatory hyperpigmentation, RCT = randomized controlled trial.

Figure 6 demonstrate asymmetric funnel plot, with multiple combination therapy data points, suggesting a publishing bias. Erythema, PIH, burning, and pruritus were observed far from the central axis. These astonishing data points support the diversity and variation of the results. Most data points exceeded an OR of 1.0, indicating a higher probability of poor combination therapy.

Forest and funnel plots indicated high variation, showing that research design, population, and interventions varied. The variability in this study may hinder its application, underlining the need for additional consistent research to resolve these contradictions.

This meta-analysis has several limitations. The studies had moderate to high heterogeneity, as indicated by significant *I*^2^ values. The study design, patient groups, and treatment resulted in heterogeneity. Laser, light treatment, and topical agent type and frequency may alter study outcomes. More conventional and detailed methodologies and consistent patient groups in future RCTs will reduce heterogeneity and improve the external validity.

Second, the funnel diagram demonstrated publication bias; however, all meta-analysis RCTs were prospectively documented and followed protocols. Study quality, sample size, and reporting methods may explain this asymmetry. Future research should prioritize large-scale, multicenter, quality-controlled investigations to reduce the residual bias.

Most adverse effects of combination therapy are minor and temporary but are more common than topical therapy. Pruritus, desquamation, and papular eruptions have high-risk ratios, necessitating close monitoring. Future RCTs should modify the dosage frequency and follow patients more closely to improve their tolerance to combination therapy.

Despite these limitations, the meta-analysis showed that laser or light therapy with topical medications can treat melasma, especially in people who do not respond well to topical treatment. This combination strategy works well and aids in the therapeutic development of melasma, supporting clinical practice and research.

### 3.6. Certainty of the evidence

The certainty of evidence for efficacy was rated as moderate, mainly downgraded due to substantial heterogeneity (*I*^2^ = 64%), despite consistent effect direction across studies. For safety outcomes, the certainty was assessed as very low due to inconsistency in AE reporting, imprecision of effect estimates (wide CIs), and suspected publication bias from selective or incomplete reporting. Both efficacy and safety outcomes were considered critically important for clinical decision-making, with importance scores of 9 and 8, respectively. A summary of these findings is provided in Appendix 3, Supplemental Digital Content, https://links.lww.com/MD/Q986.

### 3.7. Sensitivity analysis

To examine the influence of study design on efficacy, we conducted a sensitivity analysis (Appendix 4, Supplemental Digital Content, https://links.lww.com/MD/Q986) excluding 4 studies using parallel-group designs. The recalculated effect remained significant (SMD: −0.45; 95% CI: −0.65 to −0.24; *P* < .0001), and heterogeneity was notably reduced (*I*^2^ ≈ 41%), supporting the robustness of the primary findings. No sensitivity analysis was performed for safety due to the small number of studies reporting AEs and variability in AE definitions and outcomes.

## 4. Conclusion

This study supports the clinical utility of combining laser or light-based therapy with topical agents for melasma, particularly in patients who do not respond adequately to topical treatments alone. Despite the observed increase in AEs, most reactions such as erythema and PIH were self-limiting and could be managed effectively with supportive measures. Importantly, this review highlights the need for personalized treatment selection based on individual patient characteristics.

For clinical practice, the type of melasma should guide therapeutic decisions. Epidermal melasma may respond better to superficial fractional lasers combined with hydroquinone or triple combination creams, while dermal or mixed-type melasma may require gentler, repeated sessions using Q-switched Nd:YAG laser at low affluences, with adjuncts like tranexamic acid or niacinamide to prevent pigment rebound. Patients with darker skin types (Fitzpatrick IV–VI) are at greater risk for PIH and should be treated with caution, preferably using lower fluences, fewer passes, and protective post-procedure regimens including corticosteroids or anti-inflammatory agents. Lighter skin types (Fitzpatrick I–III) may tolerate more intensive protocols.

The duration and chronicity of melasma should also be considered. Long-standing or recurrent cases may benefit more from maintenance regimens involving alternating laser and topical cycles, with close follow-up. Patient education on sun protection, skincare compliance, and posttreatment monitoring is critical in reducing relapse and optimizing results.

While this study provides moderate-certainty evidence for efficacy and very low-certainty for safety outcomes, the findings remain clinically relevant. Future research should focus on stratified trials by melasma type and skin phototype, and standardized AE reporting, to develop evidence-based, individualized protocols for safer and more effective melasma treatment.

## Acknowledgments

The authors would like to express their gratitude for the funding, which was instrumental in facilitating this study.

## Author contributions

**Conceptualization:** Risha Fillah Fithria, Jinfu Peng.

**Data curation:** Risha Fillah Fithria, Yehuda Tri Nugroho Supranoto, Jinfu Peng.

**Investigation:** Risha Fillah Fithria, Yehuda Tri Nugroho Supranoto.

**Software:** Risha Fillah Fithria, Yehuda Tri Nugroho Supranoto.

**Supervision:** Zhaoqian Liu, Jinfu Peng.

**Validation:** Risha Fillah Fithria, Yehuda Tri Nugroho Supranoto, Jinfu Peng.

**Visualization:** Risha Fillah Fithria, Yehuda Tri Nugroho Supranoto.

**Writing – original draft:** Risha Fillah Fithria, Jinfu Peng.

**Writing – review & editing:** Zhaoqian Liu, Jinfu Peng.

## Supplementary Material




