# Supplementary material for: Laser and light-based therapies combined with topical agents for melasma: A systematic review and meta-analysis
Source: Medicine (Baltimore). 2026 Jan 9;105(2):e46579. doi: 10.1097/MD.0000000000046579 (PMC12795099; doi:10.1097/MD.0000000000046579)
Supplement: Supplementary file 1 [file medi-105-e46579-s001.pdf]

National Institute for Health Research

UNIVERSITY of York
Centre for Reviews and Dissemination

Systematic review

*Fields that have an **asterisk (*)** next to them means that they **must be answered. Word limits** are provided for each section. You will be unable to submit the form if the word limits are exceeded for any section. Registrant means the person filling out the form.*

This record cannot be edited because it has been marked as out of scope

1. * Review title.

Give the title of the review in English

Efficacy and Safety of Laser and Light-Based Therapies Combined with Topical Agents for Melasma: A Systematic Review and Meta-Analysis of Randomized Controlled Trials

2. Original language title.

For reviews in languages other than English, give the title in the original language. This will be displayed with the English language title.

English

3. * Anticipated or actual start date.

Give the date the systematic review started or is expected to start.

01/08/2024

4. * Anticipated completion date.

Give the date by which the review is expected to be completed.

31/08/2024

5. * Stage of review at time of this submission.

This field uses answers to initial screening questions. It cannot be edited until after registration.

Tick the boxes to show which review tasks have been started and which have been completed.

Update this field each time any amendments are made to a published record.

The review has not yet started: Yes

Review stage	Started	Completed
Preliminary searches	No	No
Piloting of the study selection process	No	No
Formal screening of search results against eligibility criteria	No	No
Data extraction	No	No
Risk of bias (quality) assessment	No	No
Data analysis	No	No

Provide any other relevant information about the stage of the review here.

6. * Named contact.

The named contact is the guarantor for the accuracy of the information in the register record. This may be any member of the review team.

Risha Fillah Fithria

Email salutation (e.g. "Dr Smith" or "Joanne") for correspondence:

Mrs Risha Fillah Fithria

7. * Named contact email.

Give the electronic email address of the named contact.

rishafithria@unwahas.ac.id

8. Named contact address

Give the full institutional/organisational postal address for the named contact.

Jl. Sriwidodo Utara II No. 295 RT.3 RW.2 Purwoyoso Ngaliyan Semarang

9. Named contact phone number.

Give the telephone number for the named contact, including international dialling code.

+6285641058202

10. * Organisational affiliation of the review.

Full title of the organisational affiliations for this review and website address if available. This field may be completed as 'None' if the review is not affiliated to any organisation.

Wahid Hasyim University OR Central South University

Organisation web address:

https://unwahas.ac.id/ OR https://en.csu.edu.cn/Schools/schools4/Pharmaceutical_Sciences

11. * Review team members and their organisational affiliations.

Give the personal details and the organisational affiliations of each member of the review team. Affiliation refers to groups or organisations to which review team members belong. **NOTE: email and country now MUST be entered for each person, unless you are amending a published record. PLEASE USE AN INSTITUTIONAL EMAIL ADDRESS IF POSSIBLE.**

Mrs Risha Fillah Fithria. 1. Wahid Hasyim University, Faculty of Pharmacy 2. Central South University, School of Pharmaceutical Sciences
Professor Zhaoqian Liu. 1) Department of Clinical Pharmacology, Hunan Key Laboratory of Pharmacogenetics, and National Clinical Research Center for Geriatric Disorders, Xiangya Hospital, Central South University, Changsha, 410008, People's Republic of China. 2) Institute of Clinical Pharmacology, Engineering Research Center for applied Technology of Pharmacogenomics of Ministry of Education, Central South University, Changsha, 410078,
Professor Jinfu Peng. Xiangya School of Pharmaceutical Sciences, Central South University, No.172 Tongzipo Road, Changsha, 410031, Hunan, China
Dr Yehuda Tri Nugroho Supranoto. Bioinformatics Research Center, Institut Bioinformatika (INBIO)-Indonesia

12. * Funding sources/sponsors.

Details of the individuals, organizations, groups, companies or other legal entities who have funded or sponsored the review.

No funding sources

Grant number(s)

State the funder, grant or award number and the date of award

No grant number

13. * Conflicts of interest.

List actual or perceived conflicts of interest (financial or academic).

None

14. Collaborators.

Give the name and affiliation of any individuals or organisations who are working on the review but who are not listed as review team members. **NOTE: email and country must be completed for each person, unless you are amending a published record.**

15. * Review question.

State the review question(s) clearly and precisely. It may be appropriate to break very broad questions down into a series of related more specific questions. Questions may be framed or refined using PI(E)COS or similar where relevant.

To evaluate the efficacy and safety of laser and light-based therapies combined with topical agents for people with melasma.

16. * Searches.

State the sources that will be searched (e.g. Medline). Give the search dates, and any restrictions (e.g. language or publication date). Do NOT enter the full search strategy (it may be provided as a link or attachment below.)

PubMed,NCBI, Google Scholar, ScienceDirect, Wiley, Embase, Cochrane

17. URL to search strategy.

Upload a file with your search strategy, or an example of a search strategy for a specific database, (including the keywords) in pdf or word format. In doing so you are consenting to the file being made publicly accessible. Or provide a URL or link to the strategy. Do NOT provide links to your search **results**.

https://www.crd.york.ac.uk/PROSPEROFILES/556539_STRATEGY_20240713.pdf

Alternatively, upload your search strategy to CRD in pdf format. Please note that by doing so you are consenting to the file being made publicly accessible.

Do not make this file publicly available until the review is complete

18. * Condition or domain being studied.

Give a short description of the disease, condition or healthcare domain being studied in your systematic review.

Melasma is a chronic skin condition characterized by the development of symmetrical, hyperpigmented patches, typically on the face. These patches are often brown or gray-brown in color. Melasma is also known as chloasma. The impact of melasma is primarily cosmetic, leading to significant emotional and psychological distress. The appearance of hyperpigmented patches can affect self-esteem and social interactions, causing individuals to feel self-conscious and anxious. The condition's chronic and recurrent nature poses treatment challenges, as it can be difficult to manage and may worsen with certain treatments if not done correctly. Additionally, the economic burden of ongoing treatments, sun protection measures, and

dermatological consultations can be substantial.

19. * Participants/population.

Specify the participants or populations being studied in the review. The preferred format includes details of both inclusion and exclusion criteria.

The population of this review are patients diagnosed with mild, moderate to severe bilaterally symmetrical melasma with all Fitzpatrick skin phototypes.

Patients were excluded if they were pregnant, breastfeeding, or planning to become pregnant during the study. Additionally, those with any skin condition at the treatment area, recent chemical peels or laser treatments (within three months), or use of depigmented topical agents on the face (within four weeks) were excluded. Patients with autoimmune diseases, hepatitis, coagulopathy, thyroid diseases treated with drugs like phenytoin, or using tranexamic acid, tretinoin, or topical skin pigmentation medications within the last month were also excluded. Exclusion criteria extended to those undergoing hormone therapy, including oral contraceptives and hormone replacement therapy, recent laser therapy for melasma (within six months), and those with a history of skin malignancy, keloid scars, immune compromise, chronic systemic diseases, active herpes lesions, or hypersensitivity to study products. Additionally, individuals on concurrent therapy affecting skin pigmentation, those with endocrine and metabolic illnesses, cutaneous infections, inflammations, ectropion, or keloidal tendencies were excluded to maintain the study's integrity and safety.

20. * Intervention(s), exposure(s).

Give full and clear descriptions or definitions of the interventions or the exposures to be reviewed. The preferred format includes details of both inclusion and exclusion criteria.

The intervention of this review is Laser or Light Therapies Combined with Topical Agents in any dose, or duration.

21. * Comparator(s)/control.

Where relevant, give details of the alternatives against which the intervention/exposure will be compared (e.g. another intervention or a non-exposed control group). The preferred format includes details of both inclusion and exclusion criteria.

The comparator in this review is topical agents that used in combination with laser or light therapies in the treatment group.

22. * Types of study to be included.

Give details of the study designs (e.g. RCT) that are eligible for inclusion in the review. The preferred format includes both inclusion and exclusion criteria. If there are no restrictions on the types of study, this should be stated.

We will exclusively include randomized controlled trials (RCTs) as they are the least biased study design for evaluating the effects of an intervention. Studies will not be excluded based on language or format.

23. Context.

Give summary details of the setting or other relevant characteristics, which help define the inclusion or exclusion criteria.

24. * Main outcome(s).

Give the pre-specified main (most important) outcomes of the review, including details of how the outcome is defined and measured and when these measurement are made, if these are part of the review inclusion criteria.

The improvement of melasma is measured using either The Melasma Area and Severity Index (MASI) score or The Modified Melasma Area and Severity Index (MASI) score.

Measures of effect

Please specify the effect measure(s) for you main outcome(s) e.g. relative risks, odds ratios, risk difference, and/or 'number needed to treat.

standardized mean differences (SMD)

25. * Additional outcome(s).

List the pre-specified additional outcomes of the review, with a similar level of detail to that required for main outcomes. Where there are no additional outcomes please state 'None' or 'Not applicable' as appropriate to the review

Adverse events

Measures of effect

Please specify the effect measure(s) for you additional outcome(s) e.g. relative risks, odds ratios, risk difference, and/or 'number needed to treat.

odds ratios (OR)

26. * Data extraction (selection and coding).

Describe how studies will be selected for inclusion. State what data will be extracted or obtained. State how this will be done and recorded.

Search results from all databases will be uploaded into Covidence/Rayyan, where two authors (RFF and YTNS) will independently screen and select records based on their titles and abstracts. Records that pass this initial screening will be identified for full-text review. The same two authors (RFF and YTNS) will then review the full text of these studies. Any disagreements regarding the inclusion of articles will be resolved through discussion and, if necessary, consultation with the senior authors (ZL and JP). All excluded records will be documented. The data extracted will include: lead author, year of publication, country, study design, setting, duration, type of melasma, Fitzpatrick skin type, sample size, intervention, control, outcome measures, effectiveness, and adverse events.

27. * Risk of bias (quality) assessment.

State which characteristics of the studies will be assessed and/or any formal risk of bias/quality assessment tools that will be used.

Two authors of this review will independently evaluate the risk of bias for each study using the Risk of Bias 2 (RoB 2) Tool from the Cochrane Handbook for Systematic Reviews of Interventions. They will resolve any discrepancies through discussion or by consulting with other authors. The risk of bias will be assessed across several domains, including bias arising from the randomization process, bias due to deviations from intended interventions, bias due to missing outcome data, bias in the measurement of the outcome, and bias in the selection of reported results. Each study will be categorized as having a 'low risk of bias', 'some concerns', or 'high risk of bias', with a justification provided for each judgment.

28. * Strategy for data synthesis.

Describe the methods you plan to use to synthesise data. This **must not be generic text** but should be **specific to your review** and describe how the proposed approach will be applied to your data. If meta-analysis is planned, describe the models to be used, methods to explore statistical heterogeneity, and software package to be used.

Meta-analyses will be conducted only if the participants, interventions, and primary outcomes are sufficiently similar to justify the combination of studies for clinical appropriateness. All outcomes, whether continuous or dichotomous data, will be analyzed quantitatively. The results will be presented as standardized mean differences (SMD) and odds ratios (OR) with 95% confidence intervals (CI), using either a random-effects model (REM) or a fixed-effects model (FEM) forest plot.

29. * Analysis of subgroups or subsets.

State any planned investigation of ‘subgroups’. Be clear and specific about which type of study or participant will be included in each group or covariate investigated. State the planned analytic approach.

The authors will assess the robustness of the study results through sensitivity analyses by repeating the primary meta-analysis and substituting alternative decisions for indistinct outcome values. The level of heterogeneity due to data variability will be evaluated to determine the feasibility of conducting subgroup analyses. Potential subgroup analyses could include follow-up time and types of adverse events. The heterogeneity level will be assessed using the I^2 statistic and the p-value from the I^2 test of heterogeneity.

The statistical tests for this meta-analysis will be conducted using Review Manager (RevMan) version 5.4. Publication bias will be evaluated using funnel plots generated by RevMan V5.4. An asymmetrical funnel plot indicates potential publication bias, while a symmetrical funnel plot suggests the absence of publication bias. The interpretation of the funnel plot will follow guidelines from the Cochrane Handbook for Systematic Reviews of Interventions.

30. * Type and method of review.

Select the type of review, review method and health area from the lists below.

Type of review

Cost effectiveness

No

Diagnostic

No

Epidemiologic

No

Individual patient data (IPD) meta-analysis

No

Intervention

No

Living systematic review

No

Meta-analysis

Yes

Methodology

No

Narrative synthesis

No

Network meta-analysis

No

Pre-clinical

No

Prevention

No

Prognostic

No

Prospective meta-analysis (PMA)

No

Review of reviews

No

Service delivery

No

Synthesis of qualitative studies

No

Systematic review
Yes

Other
No

Health area of the review

Alcohol/substance misuse/abuse
No

Blood and immune system
No

Cancer
No

Cardiovascular
No

Care of the elderly
No

Child health
No

Complementary therapies
No

COVID-19
No

Crime and justice
No

Dental
No

Digestive system
No

Ear, nose and throat
No

Education
No

Endocrine and metabolic disorders
No

Eye disorders

No

General interest

No

Genetics

No

Health inequalities/health equity

No

Infections and infestations

No

International development

No

Mental health and behavioural conditions

No

Musculoskeletal

No

Neurological

No

Nursing

No

Obstetrics and gynaecology

No

Oral health

No

Palliative care

No

Perioperative care

No

Physiotherapy

No

Pregnancy and childbirth

No

Public health (including social determinants of health)

No

Rehabilitation

No

Respiratory disorders

No

Service delivery

No

Skin disorders

Yes

Social care

No

Surgery

No

Tropical Medicine

No

Urological

No

Wounds, injuries and accidents

No

Violence and abuse

No

31. Language.

Select each language individually to add it to the list below, use the bin icon to remove any added in error.

English

There is not an English language summary

32. * Country.

Select the country in which the review is being carried out. For multi-national collaborations select all the countries involved.

Indonesia

33. Other registration details.

Name any other organisation where the systematic review title or protocol is registered (e.g. Campbell, or The Joanna Briggs Institute) together with any unique identification number assigned by them. If extracted data will be stored and made available through a repository such as the Systematic Review Data Repository (SRDR), details and a link should be included here. If none, leave blank.

34. Reference and/or URL for published protocol.

If the protocol for this review is published provide details (authors, title and journal details, preferably in Vancouver format)

Add web link to the published protocol.

Or, upload your published protocol here in pdf format. Note that the upload will be publicly accessible.

No I do not make this file publicly available until the review is complete

Please note that the information required in the PROSPERO registration form must be completed in full even if access to a protocol is given.

35. Dissemination plans.

Do you intend to publish the review on completion?

Yes

Give brief details of plans for communicating review findings.?

36. Keywords.

Give words or phrases that best describe the review. Separate keywords with a semicolon or new line. Keywords help PROSPERO users find your review (keywords do not appear in the public record but are included in searches). Be as specific and precise as possible. Avoid acronyms and abbreviations unless these are in wide use.

37. Details of any existing review of the same topic by the same authors.

If you are registering an update of an existing review give details of the earlier versions and include a full bibliographic reference, if available.

38. * Current review status.

Update review status when the review is completed and when it is published.New registrations must be ongoing so this field is not editable for initial submission.

Please provide anticipated publication date

Review_Ongoing

39. Any additional information.

Provide any other information relevant to the registration of this review.

40. Details of final report/publication(s) or preprints if available.

Leave empty until publication details are available OR you have a link to a preprint (NOTE: this field is not editable for initial submission). List authors, title and journal details preferably in Vancouver format.

Give the link to the published review or preprint.

Supplemental 3: GRADE Summary of Findings Table for primary outcomes: Efficacy (MASI and mMASI) and Safety (Adverse Events)

Author(s): Risha Fillah Fithria, Yehuda Tri Nugroho Supranoto, Zhaoqian Liu, Jinfu Peng

Question: Melasma: Laser + Topical compared to Topical only for melasma patients

Setting: Outpatient dermatology clinics / aesthetic centers

Bibliography:

Certainty assessment							№ of patients		Effect		Certainty	Importance
**№ of studies**	**Study design**	**Risk of bias**	**Inconsistency**	**Indirectness**	**Imprecision**	**Other considerations**	**Melasma: Laser + Topical**	**Topical only**	**Relative (95% CI)**	**Absolute (95% CI)**		
**MASI Score**												
11	randomised trials	not serious	serious[a]	not serious	not serious	none	699	667	-	SMD **0.55 lower** (0.74 lower to 0.36 lower)	⨁⨁⨁◯ Moderate[a]	CRITICAL
**Adverse Events**												
11	randomised trials	not serious	serious[b]	not serious	serious[c]	publication bias strongly suspected[d]	351/855 (41.1%)	140/835 (16.8%)	**OR 8.96** (3.71 to 21.64)	**476 more per 1,000** (from 260 more to 646 more)	⨁◯◯◯ Very low[b,c,d]	CRITICAL

CI: confidence interval; **OR:** odds ratio; **SMD:** standardised mean difference

Explanations

a. Moderate heterogeneity ($I^2 = 64\%$)

b. Wide variation in AE types and frequency across studies

c. Wide confidence interval (3.71 to 21.64) reflects high uncertainty

d. AE often incompletely reported or absent in small negative studies

Supplemental 4: Sensitivity analysis for Efficacy (MASI and mMASI) Outcome

Sensitivity Analysis Summary Table

Analysis	Studies Excluded	SMD (95% CI)	p-value	I^2	Interpretation
Primary Analysis (all studies)	–	−0.55 [−0.74, −0.36]	<0.00001	64%	Significant effect, moderate heterogeneity
Exclude parallel design studies	Souza, Bansal, Verma, Qu	−0.45 [−0.65, −0.24]	<0.0001	~41%	Effect remains significant, lower heterogeneity
